# Dectin-2-Dependent NKT Cell Activation and Serotype-Specific Antibody Production in Mice Immunized with Pneumococcal Polysaccharide Vaccine

**DOI:** 10.1371/journal.pone.0078611

**Published:** 2013-10-25

**Authors:** Tomomitsu Miyasaka, Yukiko Akahori, Masahiko Toyama, Namiko Miyamura, Keiko Ishii, Shinobu Saijo, Yoichiro Iwakura, Yuki Kinjo, Yoshitsugu Miyazaki, Kazunori Oishi, Kazuyoshi Kawakami

**Affiliations:** 1 Department of Medical Microbiology, Mycology and Immunology, Tohoku University Graduate School of Medicine, Sendai, Japan; 2 Division of Molecular Immunology, Medical Mycology Research Center, Chiba University, Chiba, Japan; 3 Division of Laboratory Animal, Research Institute for Biomedical Sciences, Tokyo University of Science, Tokyo, Japan; 4 Laboratory of Immune Regulation, Department of Chemotherapy and Mycoses, National Institute of Infectious Diseases, Tokyo, Japan; 5 Infectious Disease Surveillance Center, National Institute of Infectious Diseases, Tokyo, Japan; University of Delhi, India

## Abstract

Although thymus-independent type 2 antigens generally do not undergo Ig class switching from IgM to IgG, pneumococcal polysaccharide vaccine (PPV) induces the production of serotype-specific IgG. How this happens remains unclear, however. In the present study, PPV immunization induced production of IgG as well as IgM specific for a serotype 3-pneumococcal polysaccharide in the sera of wild-type (WT) mice, but this phenomenon was significantly reduced in Dectin-2 knockout (KO) mice. Immunization with PPV caused IL-12p40 production in WT mice, but this response was significantly reduced in Dectin-2KO mice. Likewise, immunization with PPV activated natural killer T (NKT) cells in WT mice but not in Dectin-2KO mice. Furthermore, administration of α-galactosylceramide, recombinant (r)IL-12 or rIFN-γ improved the reduced IgG levels in Dectin-2KO mice, and treatment with neutralizing anti-IFN-γ mAb resulted in the reduction of IgG synthesis in PPV-immunized WT mice. Transfer of spleen cells from PPV-immunized WT mice conferred protection against pneumococcal infection on recipient mice, whereas this effect was cancelled when the transferred spleen cells were harvested from PPV-immunized Dectin-2KO mice. These results suggest that the detection of PPV antigens via Dectin-2 triggers IL-12 production, which induces IFN-γ synthesis by NKT cells and subsequently the production of serotype-specific IgG.

## Introduction

*Streptococcus pneumoniae* is a leading causative bacterium of community-acquired pneumonia [1]-[3]. The risk of serious *S. pneumoniae* infection is increased in patients with a deficiency of IgG against pneumococcal capsular polysaccharides and phosphorylcholine, which facilitates opsonophagocytic killing by neutrophils [4], [5]. Immunization with 23-valent pneumococcal polysaccharide vaccine (PPV) results in a protective IgG response in vaccinated individuals who have a high risk of pneumococcal infection [6]–[8]. Thus, IgG production against pneumococcal polysaccharides is a key factor in protecting hosts from pneumococcal infection. However, it remains to be clarified how PPV causes the production of IgG.

Recently, C-type lectin receptors (CLRs), pattern recognition receptors (PRRs) for pathogen-derived polysaccharides, have garnered much attention from many investigators with respect to their role in host defense against fungal infection [9]. DC-associated C-type lectin-2 (Dectin-2), one of the CLRs, possesses a carbohydrate recognition domain (CRD) for the Ca^++^-dependent recognition of mannose [10], [11]. The triggering of Dectin-2-mediated stimulation by fungal hyphae or specific Ab leads to the activation of NF-κB and the production of proinflammatory cytokines [12], [13].

Capsular polysaccharide of *S. pneumoniae* is classified as a thymus-independent type 2 (TI-2) antigen, which does not require T cell help for the activation of B cells. TI-2 antigens generally fail to induce Ig class switching from IgM to IgG, affinity maturation and memory B cell response because of the lack of a cognate CD40/CD40L interaction between T and B cells [14], [15]. One notable exception to this is the fact that serotype-specific IgG2 is produced in individuals who receive PPV immunization [16], [17], although it remains to be clarified which cells are involved in this response.

In earlier studies by Snapper and co-workers, IFN-γ was found to stimulate the secretion of IgG3, a mouse homologue of human IgG2, by TI-2 antigen-stimulated B cells [18]. Natural killer (NK) T cells, which express both αβ T cell antigen receptors and NK cell markers, have been identified as a novel lymphocyte population that acts in the innate stages of immune responses [19]. These cells recognize glycolipid antigens, such as α-galactosylceramide (α-GalCer), in the context of CD1d molecules on dendritic cells (DCs) [20], which leads to the rapid production of IFN-γ and IL-4 [21], [22]. Kobrynski and co-workers demonstrated that NKT cells play a critical role in the Ab production caused by pneumococcal polysaccharides [23]. Recently, we reported evidence suggesting that NKT cells may be involved in the production of serotype-specific IgG after PPV immunization in clinical settings [24].

Given this background, in the present study, we addressed the question of whether Dectin-2 and NKT cells contributed to the Ab production caused by PPV immunization. We found that Dectin-2 was absolutely required for the production of IL-12p40 by dendritic cells upon in-vitro stimulation with PPV, and that immunization with PPV caused IFN-γ synthesis by NKT cells, which was possibly activated by the secretion of IL-12 from DCs leading to the production of serotype-specific Ab.

## Materials and Methods

### Mice

Dectin-2KO mice were generated by homologous recombination of the *Clec4n* gene as described previously [13]. These mice were back-crossed for seven generations to C57BL/6J. Male or female mice at 6 to 12 weeks of age were used for the experiments. Wild-type (WT) littermate mice for Dectin-2KO mice were used as controls. The experiments were approved by the ethics committees of Tohoku University (Permit Number: 2011 idou-201). We took the utmost care to alleviate any pain and suffering on the part of the mice.

### Reagents and vaccination

Twenty-three-valent pneumococcal polysaccharide vaccine (PPV; Pneumovax®NP) was purchased from MSD K.K., Tokyo, Japan. The PPV contained 25 µg each of 23 different types of pneumococcal polysaccharide antigen. WT or Dectin-2KO mice were vaccinated intraperitoneally with 20 µL of PPV diluted in 200 µL normal saline. Serum samples were collected at various time intervals post-vaccination. Alpha-GalCer was purchased from Funakoshi (Tokyo, Japan) and prepared according to the manufacturer's instructions. For in-vitro experiments, RPMI1640 medium and fetal calf serum (FCS) were obtained from Nippro (Osaka, Japan) and BioWest (Nuaillé, France), respectively. Lipopolysaccharide (LPS) prepared from *Escherichia coli* O-111 (Sigma-Aldrich, St. Louis, MO, USA) and mannan from *Saccharomyces cerevisiae* (Sigma-Aldrich) were used as a control.

### Bacteria

A serotype-3 clinical strain of *S. pneumoniae,* designated as URF918, was established from a patient with pneumococcal pneumonia [25]. The bacteria were cultured in Todd-Hewitt broth (Difco, Detroit, MI, USA) at 37°C in a 5% CO_2_ incubator, harvested at 6 h, at the mid-log phase of growth, and then washed twice in PBS. The inoculum was prepared at 3.3×10^8^ colony forming units (CFU)/ml and then stored at −80°C until use.

### Preparation and culture of dendritic cells

Bone marrow-derived dendritic cells (BM-DCs) were prepared as previously described [26]. In brief, BM cells from WT mice and Dectin-2KO mice were cultured at 2×10^5^/ml in 10 ml RPMI1640 medium supplemented with 10% FCS, 100 U/ml penicillin G, 100 µg/ml streptomycin, 2 mM L-glutamine, and 50 µM 2-mercaptoethanol (Sigma-Aldrich) containing 20 ng/ml murine granulocyte-macrophage colony-stimulating factor (GM-CSF; Wako Pure Chemical Industries, Ltd., Osaka, Japan). On day 8, non-adherent cells were collected and used as BM-DCs. BM-DCs were stimulated at 1×10^5^/ml for 24 h at 37°C in 5% CO_2_ with various concentrations of PPV or other stimuli. Methyl-α-D-mannopyranoside (ManP) (Sigma-Aldrich) was used for a competitive inhibition assay against mannose binding to Dectin-2.

### Deletion of ConA-binding fraction of PPV

In order to delete the Concanavalin A (ConA)-binding fraction, PPV was incubated with ConA Sepharose4B (GE Healthcare Bio-Sciences AB, Uppsala, Sweden) for 15 min at room temperature. The ConA unbound fraction was used to stimulate BM-DCs.

### Measurement of serotype-specific Ab and cytokines

The quantities of serotype-specific Ab against pneumococcal polysaccharide type 3 (PPS3) in sera were measured by enzyme-linked immunosorbent assay (ELISA). Microtiter plates (Nunc A/S, Roskilde, Denmark) were coated with 3 µg/ml of each polysaccharide [American Type Culture Collection (ATCC), Manassas, VA, USA] in PBS for 1 h at 37°C. Prior to testing, serum samples were diluted with an absorption buffer of 0.05% skim milk PBS to 1∶10, and incubated at room temperature for 30 min to allow the adsorption of non-specific Ab to cell wall polysaccharide (CWP: Statens Serum Institute, Copenhagen, Denmark) and PPS22F (ATCC). HRP-conjugated goat anti-mouse IgM, IgG or IgG3 antibodies (Southern Biotechnology Associates, Birmingham, AL, USA) diluted with 1∶4000 were used as detection Ab. The concentrations of IgM, IgG and IgG3 were determined based on the absorbance at 450 nm. The concentration of IL-12p40 and TNF-α in the culture supernatants was determined by ELISA using capture and biotinylated developing antibodies (BD Biosciences, Franklin Lakes, NJ, USA). The detection limit was 15 and 9.8 pg/ml, respectively. The concentration of IL-6 was assayed using an ELISA kit (BioLegend, San Diego, CA, USA) in which the detection limit was 7.8 pg/ml.

### Transfer of spleen cells and *S. pneumoniae* infection

Spleen cells were prepared from WT or Dectin-2KO mice 14 days after PPV immunization, and the obtained cells (2×10^7^/mouse) were transferred into WT mice. One day later, mice were inoculated with live *S. pneumoniae* (1.6×10^6^ CFU/mouse) at 50 µl per mouse by insertion of a 24G I.V catheter (TERUMO, Tokyo, Japan) into the trachea.

### Assay for CD69 expression and intracellular IFN-γ production

For evaluation of CD69 expression on NK, NKT and T cells, spleen cells obtained from WT or Dectin-2KO mice on day 9 after vaccination were stained with FITC-conjugated anti-CD3 (Clone 145-2C11; BioLegend), PE-conjugated anti-NK1.1 (clone PK136; BioLegend) and APC-conjugated anti-CD69 mAb (clone H1.2F3; BioLegend). The isotype-matched control IgG for each Ab was used as a reference. For intracellular IFN-γ staining, spleen cells were incubated at 4×10^5^/ml with 5 ng/ml of phorbol 12-myristate 13-acetate, 500 ng/ml of ionomycin and 2 µM of monensin (Sigma-Aldrich) for 4 hours at 37°C before the cell surface was stained. Then, cells were incubated in the presence of cytofix/cytoperm (BD Biosciences), washed twice in BD perm/wash solution and stained with FITC-conjugated anti-IFN-γ mAb (clone XMG1.2; BD Biosciences) or control rat IgG. The stained cells were analyzed using a FACS Canto II flow cytometer (BD Biosciences). Data were collected from 30,000 individual cells using parameters of forward scatter (FSC) and side scatter (SSC) to limit the lymphocyte population.

### Administration of recombinant murine IFN-γ and IL-12

Mice were injected intraperitoneally with recombinant (r)IFN-γ (PeproTech, Inc., Rocky Hill, NJ, USA) at 20,000 IU/mouse or PBS once every four days, starting on day 7 post-vaccination. In the other experiment, rIL-12 (PeproTech) or PBS was given at 0.1 µg/mouse/day via an intraperitoneal route for one week beginning on day 1 after PPV administration.

### Activation of NKT cells

To activate NKT cells, WT or Dectin-2KO mice were injected intraperitoneally with α-GalCer (1 µg/mouse) or PBS containing 0.8% dimethyl sulfoxide (DMSO) on day 7 post-vaccination.

### Neutralization of endogenous IFN-γ

Anti-IFN-γ mAb was purified from culture supernatants of hybridoma (clone R4-6A2, ATCC) using a protein G column kit (Kirkegaard & Perry Lab.). To block endogenously synthesized IFN-γ, mice were injected intraperitoneally with mAb against this cytokine at 200 µg/mouse on days 6, 7 and 10 post-vaccination. Rat IgG (ICN Pharmaceuticals, Inc., Aurora, OH, USA) was used as a control.

### Statistical analysis

Statistical analysis was conducted using JMP software (SAS Institute Inc., Cary, NC, USA) on a Windows computer. Differences between two groups were tested using two-tail analysis in the unpaired Student's t-test. Differences among three or more groups were tested using ANOVA with post-hoc analysis (Student-Newman-Keuls test). Survival data was analyzed using the Kaplan-Meier log rank test. A *p* value less than 0.05 was considered significant.

## Results

### Production of PPS3-specific Ab is reduced in Dectin-2KO mice

**Figure 1:**
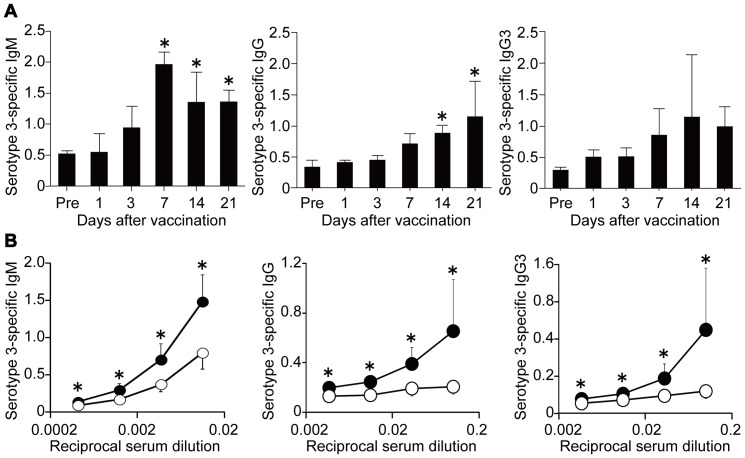
Reduced production of PPS3-specific Ab in Dectin-2KO mice. (***A***) WT mice received intraperitoneal injection of 20 µL PPV diluted in 200 µL normal saline. Sera were collected at indicated time points after PPV immunization, and concentrations of anti-PPS3 IgM, IgG and IgG3 were measured as OD450 values at×90, ×10 and×10 dilution, respectively. Data are shown as the mean±SD of four mice. *, *p*<0.05, compared with pre-vaccination level of each Ab. (***B***) Serum levels of anti-PPS-3 IgM, IgG and IgG3 on day 14 after PPV immunization were compared between WT and Dectin-2KO mice. Data are shown as the mean±SD of six mice. Similar results were obtained in three experiments. *, *p<*0.05. Closed circles, WT mice; Open circles, Dectin-2KO mice.

Initially, we measured the serum levels of anti-PPS3 IgM, IgG and IgG3 in WT mice at various time intervals after PPV immunization. As shown in Figure 1A, IgM began to increase on day 3, reached its peak level on day 7 and then decreased slightly on days 14 and 21 post-vaccination. Compared to the increase in IgM, that of IgG and IgG3 was delayed by one week. To assess the role of Dectin-2 in the Ab production caused by PPV immunization, WT and Dectin-2KO mice were injected with PPV, and the serum levels of anti-PPS3 IgM, IgG and IgG3 were measured on day 14. As shown in Figure 1B, IgM, IgG and IgG3 levels on day 14 were significantly reduced in Dectin-2KO mice compared to WT mice. Similarly, production of IgM against serotypes 6B, 14, 19F and 23F and of IgG against serotypes 6B and 19F was significantly lower in Dectin-2KO mice than in WT mice, although this difference was not observed in IgG production against serotypes 14 and 23F (see Figure S1).

### Dectin-2 is involved in the protective effect of PPV against pneumococcal infection

**Figure 2:**
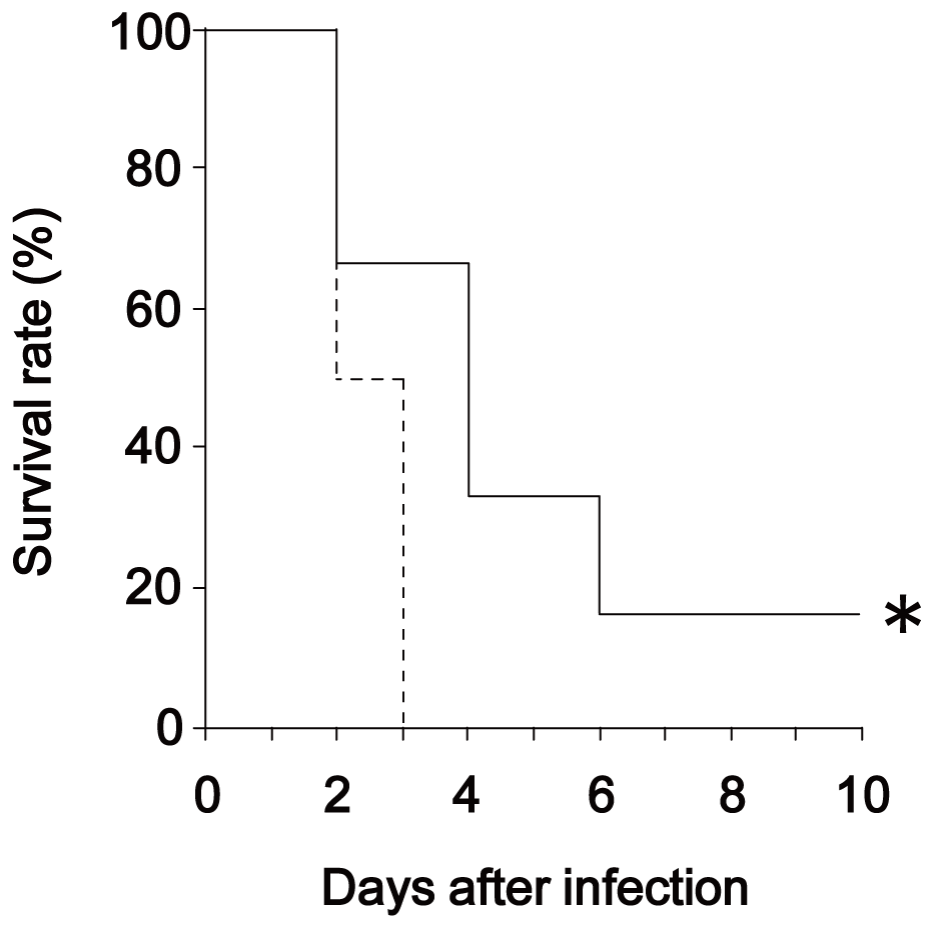
Transfer of spleen cells from PPV-immunized Dectin-2KO mice fails to induce protection from pneumococcal infection. Spleen cells obtained from WT or Dectin-2KO mice 14 days after PPV immunization were suspended in PBS. Recipient WT mice received intravenous injections of the spleen cells (2×10^7^/mouse) and, 24 h later, were infected intratracheally with *S. pneumoniae* (1.6×10^6^/mouse). The number of live mice was daily noted. Solid line, mice given spleen cells from PPV-immunized WT mice (n = 6); dotted line, mice given spleen cells from PPV-immunized Dectin-2KO mice (n = 6). Similar results were obtained in two experiments.*, *p<*0.05, compared with mice given spleen cells from PPV-immunized Dectin-2KO mice.

To elucidate whether Dectin-2 deficiency affected the host protection caused by PPV vaccination against pneumococcal infection, spleen cells were prepared from WT and Dectin-2KO mice on day 14 after vaccination and transferred into uninfected WT mice that were then infected with *S. pneumoniae* 24 h after the cell transfer (see Figure S2A). As shown in Figure 2, all of the mice that received the transfer from vaccinated Dectin-2KO mice were dead within three days post-infection; in contrast, among the mice that received the transfer from vaccinated WT mice, survival was prolonged and 17% remained alive throughout the observation period. In addition, body weight loss and hypothermia caused by pneumococcal infection were improved in the group that received spleen cells transferred from vaccinated WT mice compared to those that received spleen cells transferred from vaccinated Dectin-2KO mice (see Figure S2B).

### Role of Dectin-2 in the activation of BM-DCs upon stimulation with PPV

**Figure 3:**
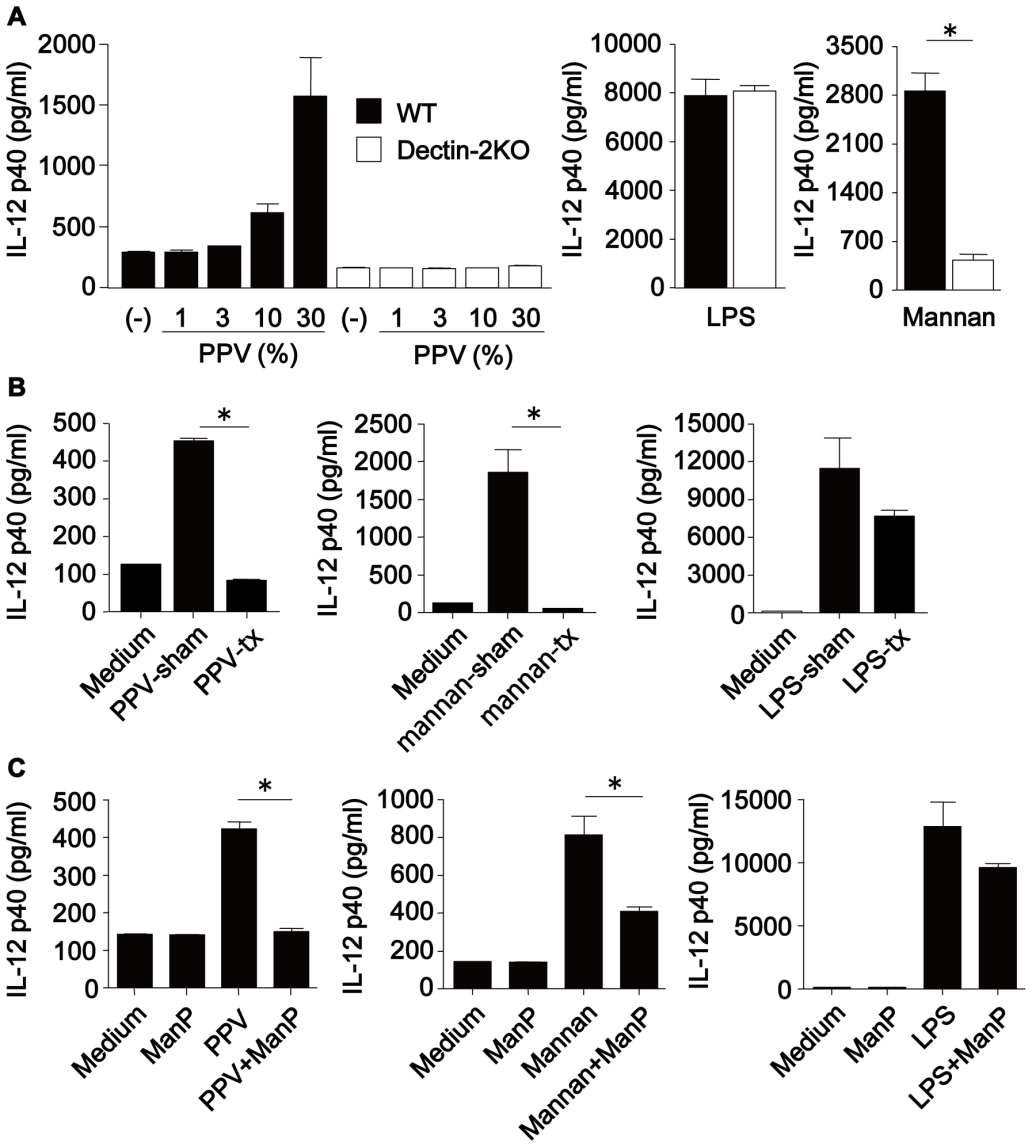
Dectin-2 is essential for PPV-induced IL-12p40 production by BM-DCs. *(* ***A*** *)* BM-DCs derived from WT or Dectin-2KO mice were cultured with indicated doses of PPV for 24 h. Concentration of IL-12p40 in the culture supernatants was measured. LPS and mannan were used at 1 µg/ml and 3 mg/ml, respectively, as controls. Data are shown as the mean±SD of triplicate cultures. Closed column, WT mice; Open column, Dectin-2KO mice. (***B***) BM-DCs derived from WT mice were stimulated with ConA-unbound fraction of PPV, mannan and LPS or sham-treated PPV, mannan and LPS for 24 h. Concentrations of IL-12p40 in the culture supernatants were measured. Data are shown as the mean±SD of triplicate cultures. PPV-sham, mannan-sham and LPS-sham: sham-treated PPV, mannan and LPS; PPV-tx, mannan-tx and LPS-tx, ConA-bound polysaccharide-deleted PPV, mannan and LPS. (***C***) BM-DCs derived from WT mice were stimulated with 30% PPV, 1 µg/ml LPS or 3 mg/ml mannan in the presence or absence of 50mM methyl-α-D-mannopyranoside (ManP) for 24 h. Similar results were obtained in three experiments.*, *p<*0.05.

To elucidate the role of Dectin-2 in the activation of immune cells by PPV, we compared the production of IL-12p40 due to stimulation with PPV in BM-DCs from WT and Dectin-2KO mice. As shown in Figure 3A, IL-12p40 synthesis by WT BM-DCs was induced by PPV in a dose-dependent fashion, whereas such production was mostly abrogated in BM-DCs from Dectin-2KO mice. A similar pattern was observed upon stimulation with mannan, the detection of which is mediated by Dectin-2 [13]. By contrast, synthesis of this cytokine by BM-DCs was not affected when cells were stimulated with LPS, which is recognized by TLR4. Similarly, production of TNF-α and IL-6 by BM-DCs upon stimulation with PPV was also abrogated in Dectin-2KO mice (see Figure S3A and B).

ConA is widely used as an α-mannose-binding lectin. To address the involvement of ConA-bound polysaccharides, we examined how depletion of the fraction bound to this lectin from PPV affected IL-12p40 synthesis by BM-DCs. As shown in Figure 3B, IL-12p40 synthesis was completely abolished by this depletion. ManP competes the binding of α-mannose to ConA. In next experiments, we examined how addition of excessive amount of ManP affected the activation of BM-DCs caused by PPV. This treatment led to the complete diminution of IL-12p40 synthesis (Figure 3C). These results suggest that Dectin-2 may sense some sugar moieties bound to ConA, such as α-mannose contained in PPV polysaccharides. In disagreement with this suggestion, however, we did not detect IL-12p40 synthesis by BM-DCs stimulated with ConA-bound fraction of PPV (data not shown).

### Involvement of IL-12 in Ab production induced by PPV immunization

**Figure 4:**
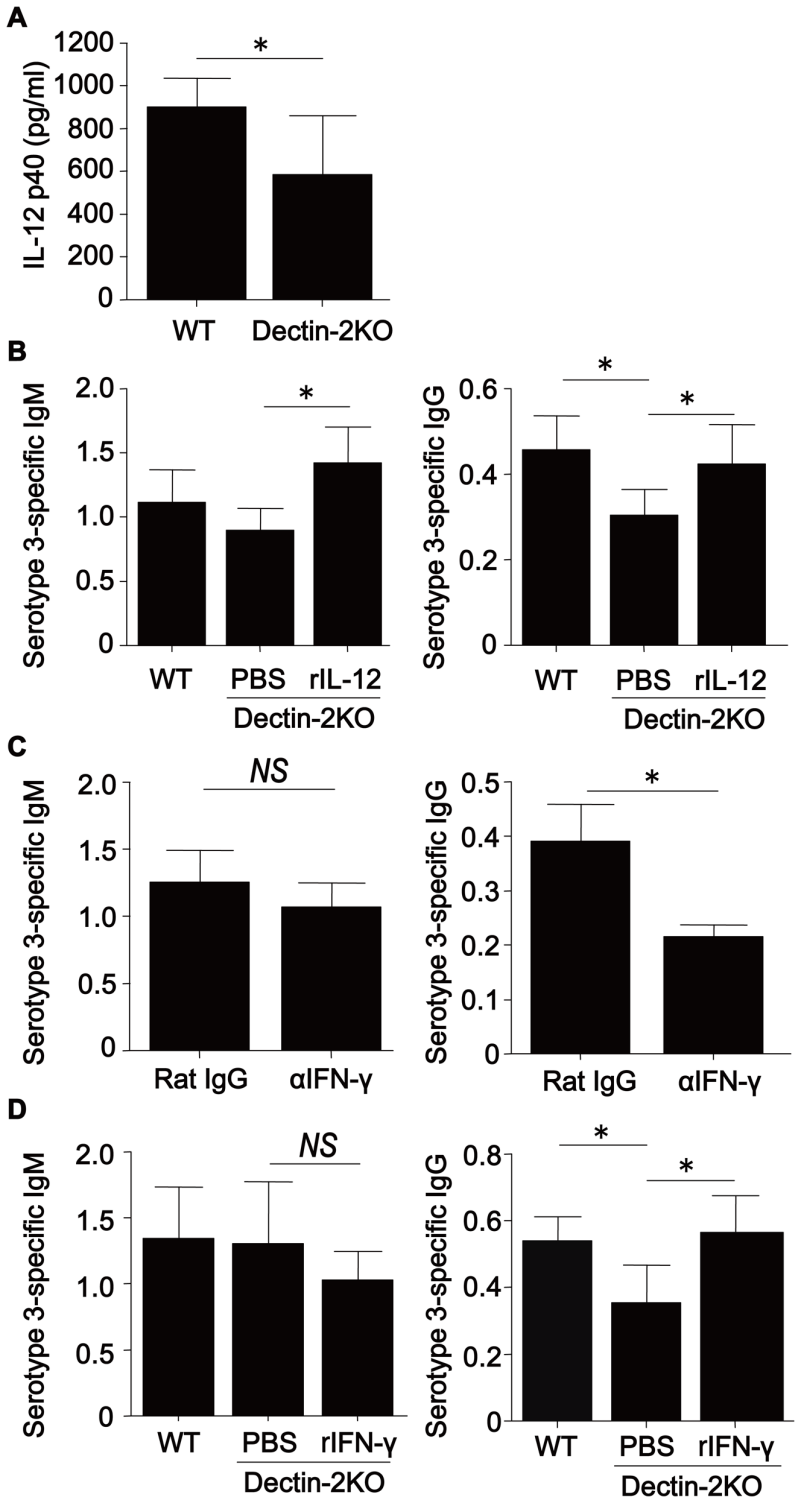
Involvement of IL-12 and IFN-γ in the production of PPS3-specific Ab after PPV immunization. (***A***) Sera were collected from WT or Dectin-2KO mice on day 9 after PPV immunization, and the concentrations of IL-12p40 were measured. Each column represents the mean±SD of five mice. (***B***) Mice received daily intraperitoneal injections of rIL-12 (0.1 µg/mouse each time) or PBS for seven days beginning on day 1 after PPV immunization. (***C***) WT mice were given intraperitoneal injections of anti-IFN-γ mAb or rat IgG (200 µg/mouse each time) on days 6, 7 and 10. (***D***) PPV-immunized Dectin-2KO mice received daily intraperitoneal injections of rIFN-γ (20,000 IU/mouse each time) or PBS for four days, starting on day 7 post-PPV immunization. On day 14, serum levels of IgM and IgG were measured as OD450 values at×90 and×30 dilution, respectively. Data are shown as the mean±SD of five to six mice. Similar results were obtained in two experiments.*, *p*<0.05; *NS*, not significant.

To address this possibility, we measured the serum concentration of IL-12p40 on day 9 after PPV immunization. As shown in Figure 4A, IL-12p40 levels were lower in Dectin-2KO mice than in WT mice. These results suggest that the impaired Ab production was due to attenuated IL-12 synthesis in Dectin-2KO mice; accordingly, we next asked whether the administration of rIL-12 altered the production of Ab in Dectin-2KO mice. As shown in Figure 4B, this treatment completely improved the reduced production of anti-PPS3 IgM and IgG in Dectin-2KO mice, which was consistent with this hypothesis.

### Involvement of IFN-γ in Ab production induced by PPV immunization

To elucidate the possible involvement of IFN-γ in Ab production induced by PPV immunization and mediated by Dectin-2, we first examined the effect of treatment with neutralizing mAb against this cytokine on the production of anti-PPS3 Ab in WT mice. As shown in Figure 4C, this treatment significantly reduced the production of anti-PPS3 IgG, but not of IgM, on day 14 compared to treatment with control rat IgG. In addition, we sought to establish whether the administration of rIFN-γ altered the production of Ab in Dectin-2KO mice. As shown in Figure 4D, this treatment completely improved the reduced production of anti-PPS3 IgG in Dectin-2KO mice, which was consistent with this hypothesis.

### Involvement of NKT cells in Ab production induced by PPV immunization

**Figure 5:**
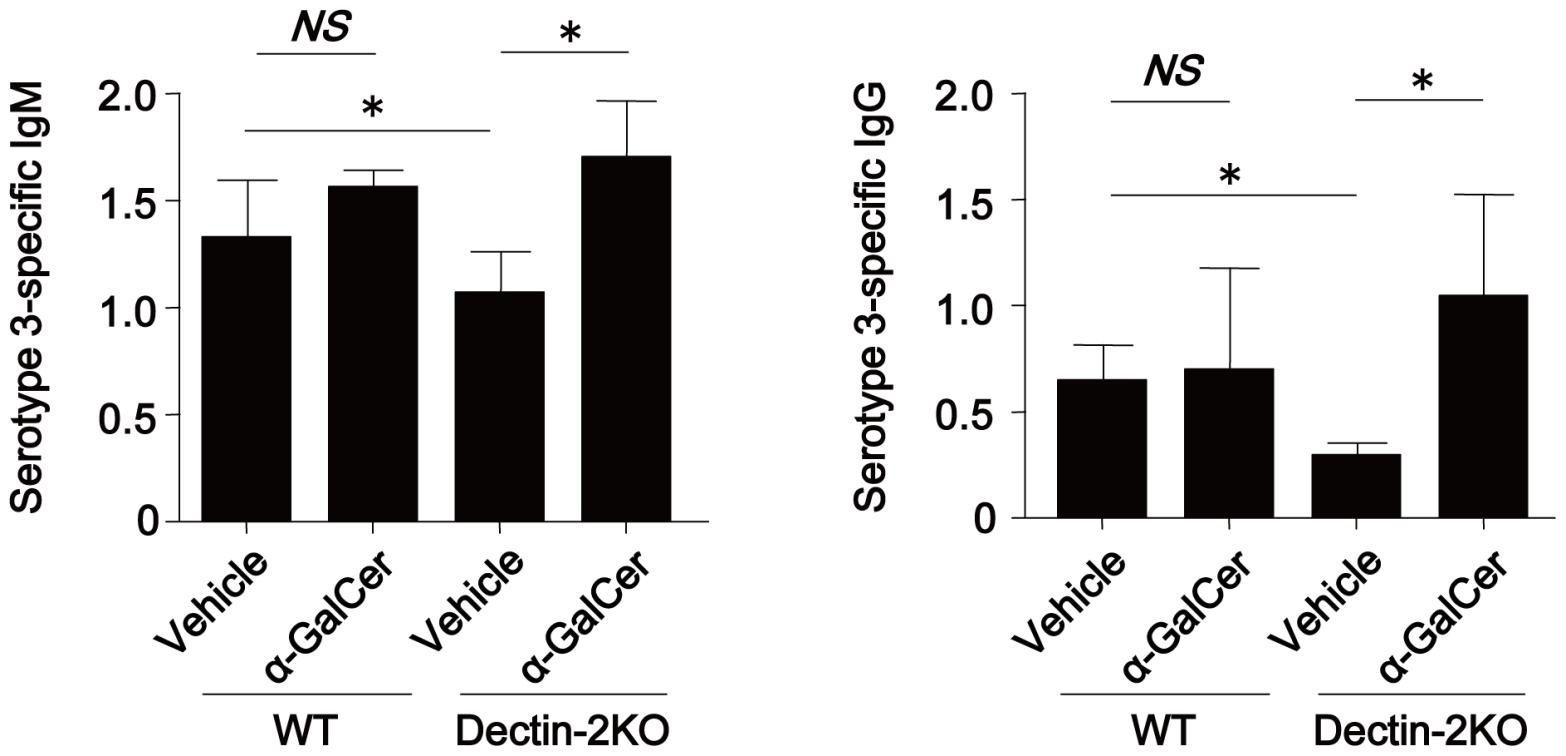
Effect of α-GalCer treatment on the reduced production of PPS3-specific Ab in Dectin-2KO mice. Sera were collected from WT or Dectin-2KO mice on day 14 after PPV immunization. These mice received an intraperitoneal injection of α-GalCer (1 µg/mouse) or vehicle on day 7 post-PPV immunization. Concentrations of PPS3-specific IgM and IgG in sera were measured as OD450 values at×90 and×10 dilution, respectively. Data are shown as the mean±SD of four mice. Similar results were obtained in two experiments.*, *p*<0.05.

**Figure 6:**
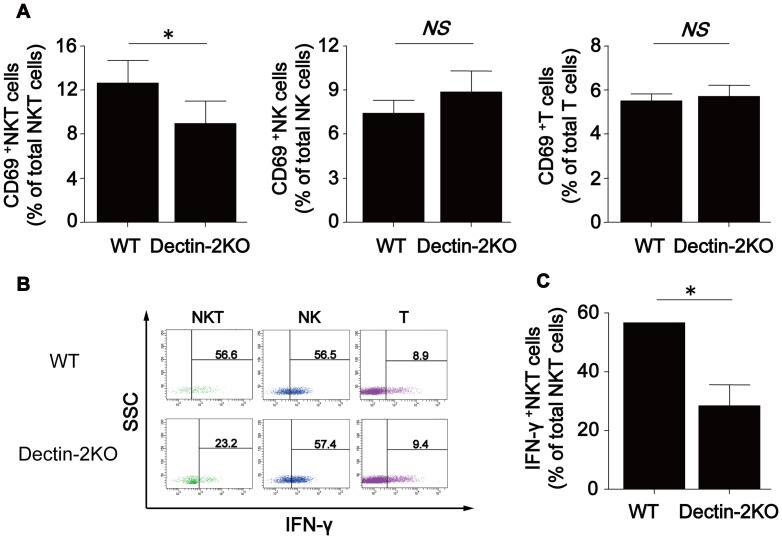
NKT cell activation by PPV immunization. Spleen cells were prepared from WT and Dectin-2KO mice on day 9 after PPV immunization. (***A***) The obtained cells were stained with FITC-conjugated anti-CD3, PE-conjugated anti-NK1.1, and APC-conjugated anti-CD69 mAbs. Expression of CD69 in NKT (CD3^+^NK1.1^+^), NK (CD3^-^NK1.1^+^) and T (CD3^+^NK1.1^-^) cells were analyzed using flow cytometer. Data are shown as the mean±SD of five mice. *, *p*<0.05; *NS*, not significant compared with WT mice. (***B***) Intracellular expression of IFN-γ in NKT, NK and T cells was analyzed using flow cytometer. Cut-off lines were determined on the basis of isotype-matched control IgG profile. Representative profile of the cytokine expression in each subset is shown. (***C***) Percent of IFN-γ^+^ population in NKT cells was analyzed in each group. Data are shown as the mean±SD. Similar results were obtained in three experiments. *, *p*<0.05; *NS*, not significant.

Finally, in order to elucidate the possible involvement of NKT cells in Ab production resulting from PPV immunization and mediated by Dectin-2, we initially asked if α-GalCer treatment altered the production of anti-PPS3 Ab in Dectin-2KO mice. As shown in Figure 5, this treatment recovered the reduced production of IgM and IgG on day 14 post-vaccination in Dectin-2KO mice. Next, we compared the expression of CD69 in splenic NKT, NK and T cells obtained from WT and Dectin-2KO mice on day 9. As shown in Figure 6A, the proportion of CD69^+^ NKT cells was lower in Dectin-2KO mice than in WT mice, whereas this difference was not observed in NK and T cells. In addition, we examined the intracellular expression of IFN-γ in splenic NKT, NK and T cells on the same day (Figure 6B), and the proportion of IFN-γ^+^ NKT cells was reduced in Dectin-2KO mice compared to WT mice (Figure 6C). These results suggest that IFN-γ secreted from NKT cells contributes to the Ab production induced by PPV immunization.

## Discussion

In the present study, we measured the serotype 3-specific Ab and used this type of *S. pneumoniae* strain isolated from a patient with pneumonia. We have demonstrated, with this bacterial strain, that NKT cells play a critical role in the innate phase of host protection against pneumococcal infection [25], [27]. Briles and co-workers demonstrated that the median time to death in mice which died of infection with the serotype 3 or 4 strain was significantly shorter than the other pneumococcal types [28]. Furthermore, a recent article reported that serotype 3 was highly correlated with case-fatality ratios in invasive pneumococcal infection [29]. Thus, serotype 3 pneumococcus is one of the clinically important bacteria in this infection [30]. In our further experiments, production of IgM against PPS6B, 14, 19F and 23F as well as PPS3 was significantly reduced in Dectin-2KO mice compared to WT mice, as was IgG production against PPS6B and 19F but not against PPS14 and 23F (see Figure S1), suggesting that not all polysaccharides in PPV may be detected solely through Dectin-2. In this respect, PPS3, 6B and 19F are reported to have relatively simple structures without side chains, in contrast to the more complicated structures of 14 and 23F [31]; the simpler structures might be related to the different effects of Dectin-2-deficiency on Ab responses. Furthermore, in an in-vitro binding assay, DC-SIGN interacts with *S. pneumoniae* serotypes 3 and 14, but not certain other serotypes including 19F [32]. SIGN-R1 plays an essential role in eliciting the host immune response and protecting against infection with serotypes 2 and 14 *S. pneumoniae* by mediating recognition and clearance of these bacteria [33], [34], although it is not directly involved in PPS-specific production of IgM and IgG [35]. These findings suggest that the binding specificity of each PPS to Dectin-2 may not be uniform.

Earlier investigations reported an important role of IL-12 in the host defense against pneumococcal infection. Exogenous administration of IL-12 enhanced the innate immune response in lungs against *S. pneumoniae* infection by inducing IFN-γ production [36] and increased the host protection against this infection [37], [38]. Furthermore, in a study by Buchanan and co-workers, co-administration of IL-12 enhanced anti-PPS IgG production caused by PPV conjugated to diphtheria toxin CRM_197_ [39]. We also previously showed that IL-12p40KO mice were highly susceptible to *S. pneumoniae* infection, which was due to the reduced production of IFN-γ [40]. Thus, IL-12 has been identified as a key cytokine. We measured the serum level of IL-12p40 after PPV immunization because it was difficult to directly measure this cytokine at the local site where DCs were activated. This level was significantly reduced in Dectin-2KO mice compared with WT mice, although the reduction was less marked than we had expected based on the results of in-vitro experiments using BM-DCs, in which IL-12p40 production was completely abrogated in Dectin-2KO mice. In addition, replenishment of rIL-12 resulted in significant improvement of the reduced IgG production in Dectin-2KO mice. Although possible involvement of IL-23 that shares IL-12p40 [41] is not completely excluded, the current data suggest that IL-12 produced by DCs in a Dectin-2-dependent fashion may be involved in the Ab response caused by PPV immunization.

IL-12 promotes the production of IFN-γ by various cells including NKT, NK and T cells [42], [43]. Buchanan and co-workers demonstrated that IL-12 enhances the Ab response to thymus-independent polysaccharide antigens in the absence of T and NK cells [44]. In our clinical study, NKT cells were suggested to contribute to the production of serotype-specific IgG in humans after PPV immunization [24]. Furthermore, the role of NKT cells in supporting the proliferation and production of Ab by naïve and memory B cells has been extensively investigated [45]-[47]. Kobrynski and co-workers demonstrated that CD1d-deficient mice are impaired in the production of IgG specific for pneumococcal capsular polysaccharides, but not for a protein antigen [23]. These earlier findings raise the possibility that the detection of PPV polysaccharides via Dectin-2 may lead to the activation of NKT cells, which contribute to the PPV-induced Ab response through the secretion of IFN-γ. In keeping with this possibility, we observed activation of NKT cells, as evidenced by an increased expression of CD69 and the intracellular production of IFN-γ, in the spleen after PPV immunization; their activation was reduced in the absence of Dectin-2. In further experiments, the replenishment of rIFN-γ improved the reduced Ab production caused by PPV in Dectin-2KO mice and the administration of neutralizing anti-IFN-γ mAb led to an attenuated Ab response in vaccinated WT mice. Interestingly, Snapper and co-workers previously demonstrated that IFN-γ strongly induces the production of IgG3 by B cells upon stimulation with a thymus-independent type-2 antigen [48]. In addition, mice lacking IgG3 are susceptible to *S. pneumoniae* infection and not protected from this infection by immunization with PPS [49], suggesting that IFN-γ may be involved in class switching of Ab to IgG under these conditions. In agreement with these earlier observations, production of PPS3-specific IgG3 was almost completely abolished in Dectin-2KO mice compared to WT mice. Considered together with the findings in previous investigations, the results of the present study indicate that IFN-γ secreted from NKT cells as a downstream event of Dectin-2-mediated DC activation plays an important role in serotype-specific IgG production after PPV immunization.

To the best of our knowledge, these data are the first evidence showing that Dectin-2 is involved in IgM and IgG production against pneumococcal capsular polysaccharides after PPV immunization. It should be noted that Ab production against pneumococcal polysaccharides was accompanied by synthesis of IL-12p40 and activation of splenic NKT cells and their production of IFN-γ in a Dectin-2-dependent fashion. These observations have important implications for understanding the precise mechanism of PPV's effects and for further improvement in the clinical effectiveness of this vaccine. Further investigations are necessary to establish in greater detail the mechanism of NKT cell activation during the Ab response after vaccination.

## Supporting Information

Figure S1 **Production of PPS6B, 14, 19F and 23F-specific Ab.** WT and Dectin-2KO mice received intraperitoneal injections of 20 µL PPV diluted in 200 µL normal saline. Sera were collected on day 14 after PPV immunization, and concentrations of anti-PPS6B, 14, 19F and 23F IgM and IgG were measured as OD450 values at ×90 and ×30 dilution, respectively. Data are shown as the mean±SD of six mice. Similar results were obtained in three experiments. *, *p*<0.05; *NS*, not significant.
(TIF)

Figure S2 **Effect of spleen cell transfer on body weight and temperature after pneumococcal infection.** (*A*) Schematic diagram of the spleen cell transfer experiment. Spleen cells were prepared from three WT or Dectin-2KO mice on day 14 after PPV immunization, and the obtained cells were transferred at 2×10^7^/mouse to six WT mice each group. One day later, the recipient WT mice were infected intratracheally with *S. pneumoniae* (1.6×10^6^ CFU/mouse). (***B***) The body weight and temperature of each mouse were measured daily. Body weight is expressed as a relative value to that before infection. Solid line, mice given spleen cells from PPV-immunized WT mice; dotted line, mice given spleen cells from PPV-immunized Dectin-2KO mice. Similar results were obtained in two experiments.
(TIF)

Figure S3 **Dectin-2 is essential for PPV-induced TNF-α and IL-6 production by BM-DCs.** BM-DCs derived from WT or Dectin-2KO mice were cultured with indicated doses of PPV for 24 h. Concentrations of TNF-α (A) and IL-6 (B) in the culture supernatants were measured. LPS and mannan were used at 1 µg/ml and 3 mg/ml, respectively, as controls. Data are shown as the mean±SD of triplicate cultures. Similar results were obtained in three experiments. *, *p<*0.05. Closed column, WT mice; Open column, Dectin-2KO mice.
(TIF)
