# Supplementary figures and images for: Dectin-2-Dependent NKT Cell Activation and Serotype-Specific Antibody Production in Mice Immunized with Pneumococcal Polysaccharide Vaccine
Source: PLoS One. 2013 Oct 25;8(10):e78611. doi: 10.1371/journal.pone.0078611 (PMC3808275; doi:10.1371/journal.pone.0078611)

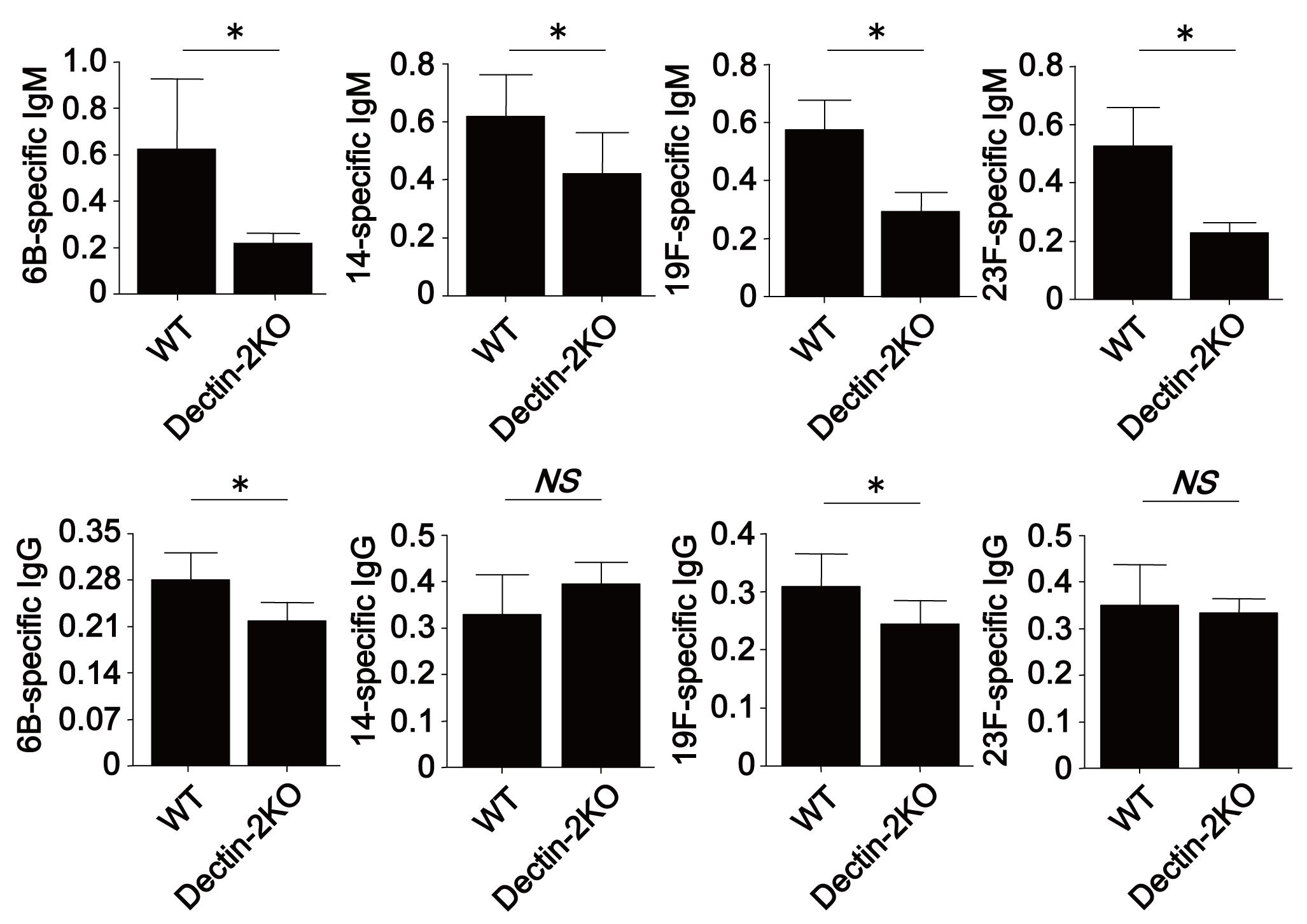

Supplement: Figure S1 — Production of PPS6B, 14, 19F and 23F-specific Ab. WT and Dectin-2KO mice received intraperitoneal injections of 20 µL PPV diluted in 200 µL normal saline. Sera were collected on day 14 after PPV immunization, and concentrations of anti-PPS6B, 14, 19F and 23F IgM and IgG were measured as OD450 values at ×90 and ×30 dilution, respectively. Data are shown as the mean±SD of six mice. Similar results were obtained in three experiments. *, p<0.05; NS, not significant. (TIF) [file pone.0078611.s001.tif]

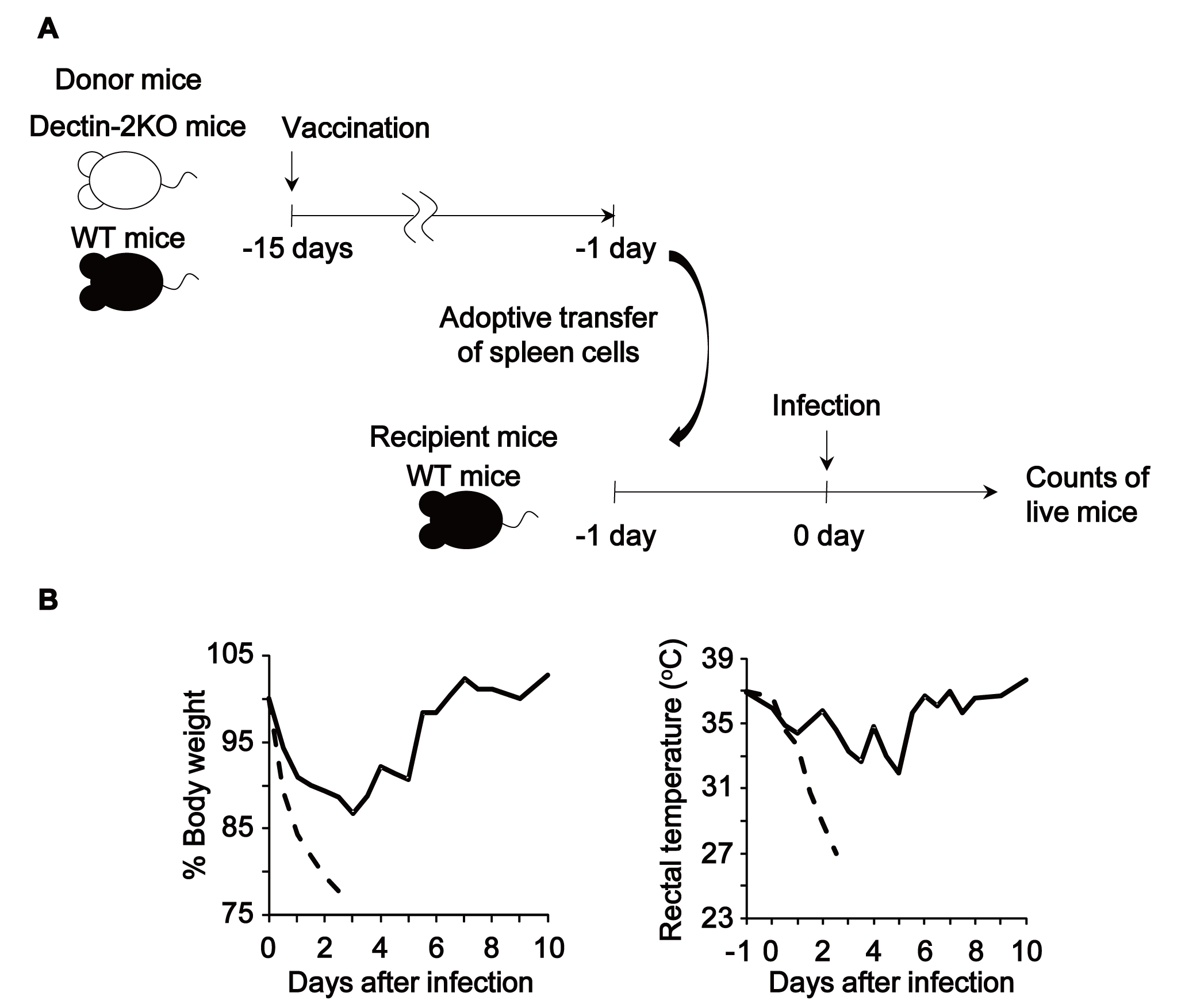

Supplement: Figure S2 — Effect of spleen cell transfer on body weight and temperature after pneumococcal infection. (A) Schematic diagram of the spleen cell transfer experiment. Spleen cells were prepared from three WT or Dectin-2KO mice on day 14 after PPV immunization, and the obtained cells were transferred at 2×10^7^/mouse to six WT mice each group. One day later, the recipient WT mice were infected intratracheally with S. pneumoniae (1.6×10^6^ CFU/mouse). (B) The body weight and temperature of each mouse were measured daily. Body weight is expressed as a relative value to that before infection. Solid line, mice given spleen cells from PPV-immunized WT mice; dotted line, mice given spleen cells from PPV-immunized Dectin-2KO mice. Similar results were obtained in two experiments. (TIF) [file pone.0078611.s002.tif]

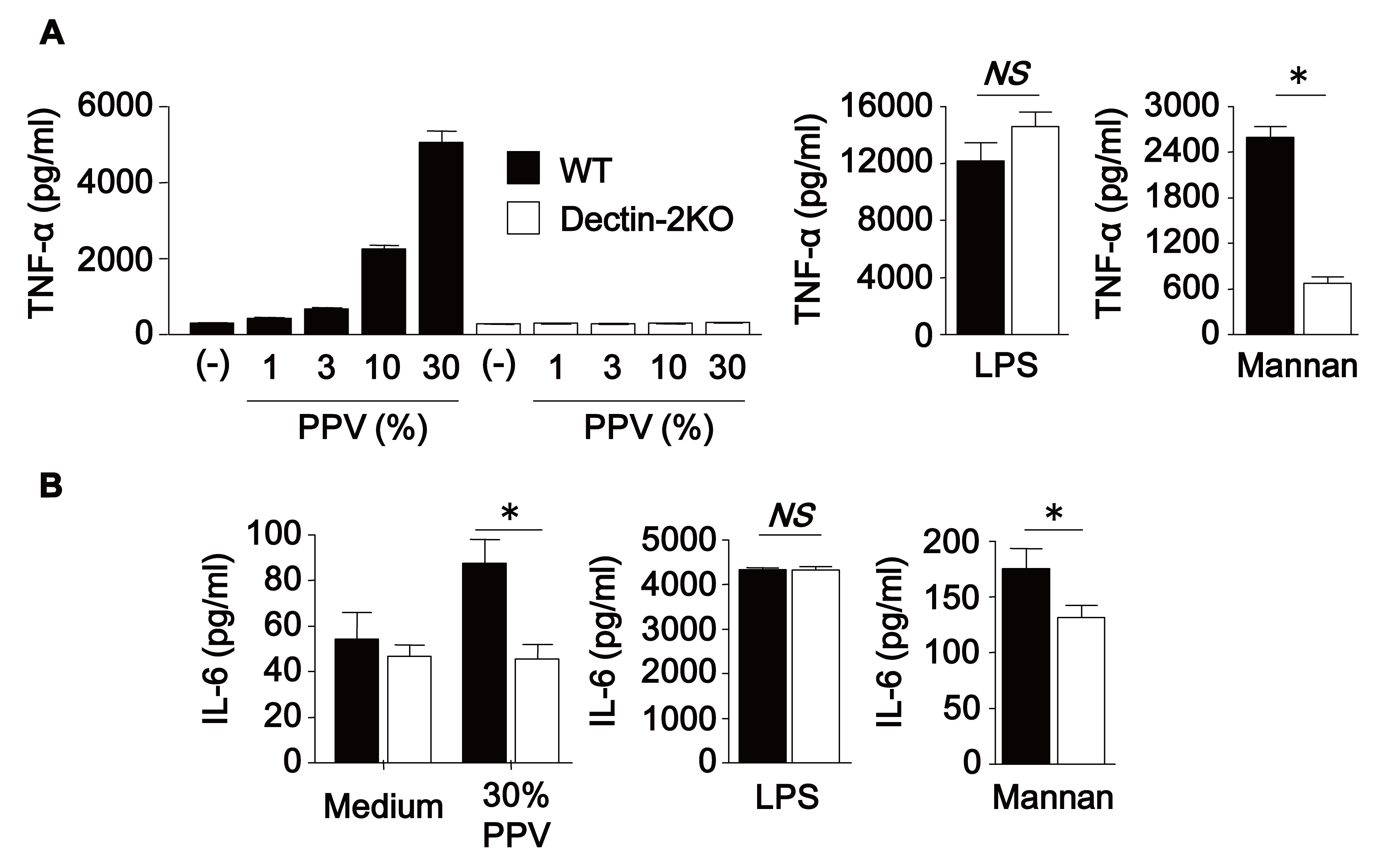

Supplement: Figure S3 — Dectin-2 is essential for PPV-induced TNF-α and IL-6 production by BM-DCs. BM-DCs derived from WT or Dectin-2KO mice were cultured with indicated doses of PPV for 24 h. Concentrations of TNF-α (A) and IL-6 (B) in the culture supernatants were measured. LPS and mannan were used at 1 µg/ml and 3 mg/ml, respectively, as controls. Data are shown as the mean±SD of triplicate cultures. Similar results were obtained in three experiments. *, p<0.05. Closed column, WT mice; Open column, Dectin-2KO mice. (TIF) [file pone.0078611.s003.tif]
